# Photosynthetic apparatus of *Rhodobacter sphaeroides* exhibits prolonged charge storage

**DOI:** 10.1038/s41467-019-08817-7

**Published:** 2019-02-22

**Authors:** Sai Kishore Ravi, Piper Rawding, Abdelnaby M. Elshahawy, Kevin Huang, Wanxin Sun, Fangfang Zhao, John Wang, Michael R. Jones, Swee Ching Tan

**Affiliations:** 1 Department of Materials Science and Engineering, National University of Singapore, 9 Engineering Drive 1, Singapore, 117575 Singapore; 2 Department of Materials Science and Engineering, University of Wisconsin-Madison, 1509 University Ave, Madison, WI 53706 USA; 3 Department of Materials Science and Engineering, University of Illinois at Urbana-Champaign, 1304W Green St, Urbana, IL 61801 USA; 4 Bruker Nano Surface Division, 11 Biopolis Way #10-10, The Helios, Singapore, 138667 Singapore; 5 School of Biochemistry, University of Bristol, Biomedical Sciences Building, University Walk, Bristol, BS8 1TD UK

## Abstract

Photosynthetic proteins have been extensively researched for solar energy harvesting. Though the light-harvesting and charge-separation functions of these proteins have been studied in depth, their potential as charge storage systems has not been investigated to the best of our knowledge. Here, we report prolonged storage of electrical charge in multilayers of photoproteins isolated from *Rhodobacter sphaeroides*. Direct evidence for charge build-up within protein multilayers upon photoexcitation and external injection is obtained by Kelvin-probe and scanning-capacitance microscopies. Use of these proteins is key to realizing a ‘self-charging biophotonic device’ that not only harvests light and photo-generates charges but also stores them. In strong correlation with the microscopic evidence, the phenomenon of prolonged charge storage is also observed in primitive power cells constructed from the purple bacterial photoproteins. The proof-of-concept power cells generated a photovoltage as high as 0.45 V, and stored charge effectively for tens of minutes with a capacitance ranging from 0.1 to 0.2 F m^−2^.

## Abstract (web summary)

Photosynthetic proteins are used to harvest solar energy in bio-photovoltaics, but are typically not investigated for charge storage. Here the authors report prolonged charge storage in multilayers of photoproteins as well as a proof-of-principle biophotonic power cell with purple bacterial photoproteins.

## Introduction

Highly quantum-efficient biophotonic processes that occur in natural photosynthetic proteins have a huge implication for future energy technologies. In a general sense, the term biophotonic process refers to a phenomenon related to the interaction of biological matter with photons^1^. Some of the most widespread and impactful examples are the photophysical and photochemical processes that take place during the initial steps of photosynthesis^2–4^. These involve the interaction of light-harvesting complexes with incoming photons to form pigment excited electronic states, ultrafast (picosecond time scale) energy transfer to efficiently move excited state energy to a reaction centre (RC) pigment–protein, and trapping of energy through photochemical charge separation^2–4^.

Besides being the core biophysical processes in nature, the processes of energy harvesting, conversion, and storage underlie many existing energy technologies^5–7^. In energy research, these three processes are usually realized separately through a modular device architecture^8^, with each requiring optimization and effective interfacing to achieve a high overall efficiency^9^. Typically, solar, thermal, chemical, or mechanical energy harvested through a variety of mechanisms is converted into electrical energy and, if not used directly, stored using a capacitor or a battery^10,11^. As there are losses involved at each stage, considerable efforts have gone into the development of hybrid systems where two or more of the three processes are combined. In some cases, energy harvesting and conversion systems might be one and the same, but devices typically have a distinct system performing the energy storage function. An emerging example of a hybrid system is the “self-charging” power cell or supercapacitor, the basic function of which is to harvest and convert ambient energy into electricity and continuously charge a battery or capacitor to ensure a sustainable supply of power^12,13^. The majority of these power cells are based on piezoelectric^14,15^ or triboelectric^16–18^ nanogenerators that harvest ambient (bio)mechanical energy and store converted energy in supercapacitors or batteries. Fewer attempts have been made to develop self-charging power cells that harvest light energy, these mainly involving the hybridization of dye-sensitized solar cells with supercapacitors^19,20^. Hybrid devices have also been described in which Li-ion batteries are photocharged by perovskite solar cells^21^, and recently a hybrid self-charging power cell was reported that combined a photoelectrochemical cell with a redox-flow battery^22^. These hybrid power cells typically contain a light-harvesting/conversion system and an electrical energy storage system combined into a single-device architecture, sometimes with a common electrode.

On studying the light harvesting and charge transport properties of a purple bacterial photosynthetic protein, multilayers of the protein are able to store the photo-generated charges for a long time spanning tens of minutes. Investigating the origin of this phenomenon and demonstrating the implication this can have for energy research formed the basis of this work. Microscopic and macroscopic evidence of this phenomenon are presented using Kelvin-probe force microscopy (KPFM), scanning capacitance microscopy (SCM), photochronopotentiometry, cyclic voltammetry (CV), and galvanostatic charge–discharge studies.

**Fig. 1:**
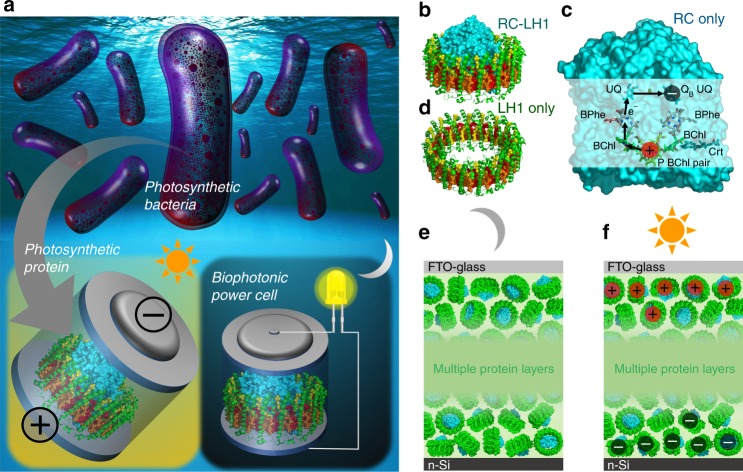
Photosynthetic proteins, charge separation, and cell architecture. **a** Cartoon illustrating the concept of a biophotonic power cell based on natural photosynthetic components. **b** The RC-LH1 complex comprises a central reaction centre (RC) charge separation domain (cyan) surrounded by a cylindrical light harvesting (LH1) domain. **c** The RC can be isolated as a separate complex and is shown as a solid object with a transparent surface in the plane of the membrane to reveal the electron transfer cofactors. Photoexcitation of the P bacteriochlorophyll (BChl) pair initiates a four-step charge separation via a BChl, bacteriopheophytin (BPhe) and ubiquinone (UQ) to reduce the Q_B_ UQ on the opposite side of the photosynthetic membrane. **d** The LH1 cylinder can be isolated as a separate complex and is formed from 32 BChls (alternating red/orange) and 32 carotenoids (yellow) held in place by a protein scaffold (green). **e** Assembled cells comprised a multilayer of purified RC-LH1 proteins (green LH1 with cyan RC) sandwiched between n-Si and FTO-glass electrodes. **f** Under illumination a photovoltage is produced due to light-activated protein/electrode redox interactions, but current does not flow through the protein multilayer in the absence of mobile mediators

Exploiting the “prolonged charge storage” behavior, we construct photoprotein-based biophotonic power cells (BPCs, Fig. 1a) in which the light harvesting, energy conversion, and charge storage processes are integrated within a single, multifunctional material. This has been realized through the use of a pigment–protein complex from a natural bacterial photosystem that absorbs energy across the solar spectrum and transiently stores harvested energy through charge separation^23–26^. Such photosystems either in the form of isolated protein complexes or as whole bacterial cells are increasingly studied for biophotovoltaic and bioelectronic applications^27–35^. The particular photoprotein used, the so-called PufX-deficient RC-light harvesting 1 (RC-LH1) complex from the photosynthetic bacterium *Rhodobacter* (*Rba*.) *sphaeroides* (Fig. 1b), is an integral membrane pigment–protein made up from a central RC domain (Fig. 1b, c, cyan) surrounded by a cylindrical LH1 antenna complex (Fig. 1b, d)^23,24,36,37^. Following the absorption of solar energy by the bacteriochlorophylls (BChls) and carotenoids of the LH1 domain (Fig. 1d, and Supplementary Fig. 1), the key energy conversion event is a four-step electron transfer between a pair of BChl molecules (P) at one end of the RC protein and a ubiquinone molecule (Q_B_) at the opposite end (Fig. 1c)^25,26^. This forms a charge-separated state (P^+^Q_B_^−^) around 1 μs after photoexcitation that is stable for a few seconds (Fig. 1c). To fabricate a simple device that generates and stores the photogenerated charges, films of concentrated photoprotein of varying thickness are sandwiched between a transparent fluorine-doped tin oxide (FTO) glass front-electrode and an n-doped silicon (n-Si) back electrode (Fig. 1e, f). We demonstrate that the resulting BPC carries out solar energy harvesting, energy conversion, and energy storage in a single, integrated architecture.

## Results

### Construction and photoresponse of a BPC

BPCs were assembled by drop casting a 20–1000 µL aliquot of concentrated RC-LH1 protein solution (see Materials and methods) into a well formed from one or more layers of plastic paraffin film (Parafilm M) adhered to an FTO-glass slide. This enabled formation of a protein multilayer of regular area and thicknesses between 0.1 and 2 mm. After partially drying the protein under a vacuum a precleaned n-type silicon counter electrode was sandwiched with the protein-coated FTO electrode and the cell was sealed. The result was a densely packed protein multilayer formed in the absence of any additional electrolyte to act as a charge carrier. For reference, a single RC-LH1 complex has a maximum diameter of ~13 nm,^23^ which means that a closely packed, 0.1 mm-thick multilayer should be the equivalent of a minimum of ~7700 stacked protein monolayers, and ~154,000 monolayers for a 2 mm film. The absorbance characteristics of RC-LH1 complexes in solution are shown in Supplementary Fig. 1.

**Fig. 2:**
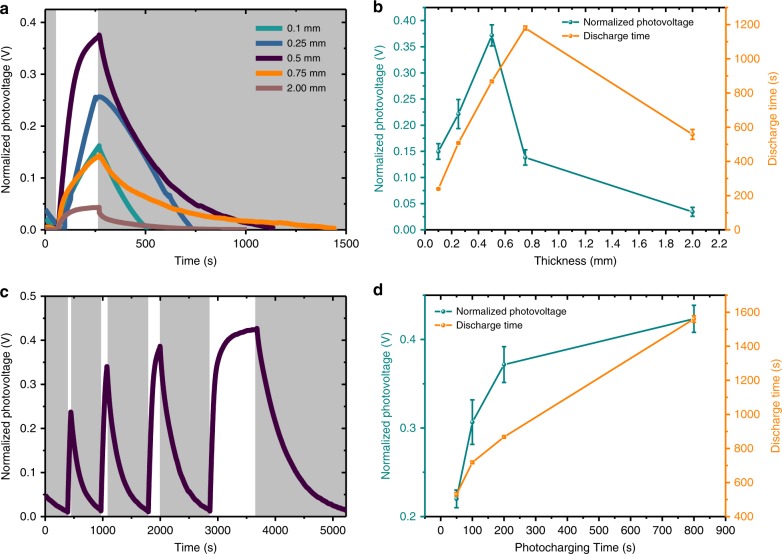
Charging and discharging of biophotonic power cells. **a**, **b** Effect of protein film thickness on photocharging and discharging in biophotonic power cell (BPC) with RC-LH1 (reaction centre-light harvesting 1 complex). **c**, **d** Effect of photocharging time on the photovoltage and discharge time in 500 μm RC-LH1 BPCs. In **a** and **c**, gray = light-off and white = light-on. In **b** and **d**, data points are the average values from three replicates with error bars representing a standard deviation

Five RC-LH1 BPCs of varying thickness were charged by exposure to 1 sun illumination for 200 s under open-circuit conditions. The photovoltage achieved increased with the thickness of the protein multilayer up to 500 µm, beyond which the maximum photovoltage dropped (Fig. 2a). In addition to the highest photovoltage under this standard illumination period (≈0.37 V; Fig. 2b), the 500 µm film exhibited the second longest dark discharge time (Fig. 2b). For these 500 µm cells, the photovoltage increased as the photocharging time was increased (Fig. 2c, d), as did the dark discharge time. The longest discharge time obtained was 1560 s (26 min) for a charging time of 800 s (Fig. 2d).

### Origin of the voltage build-up

**Fig. 3:**
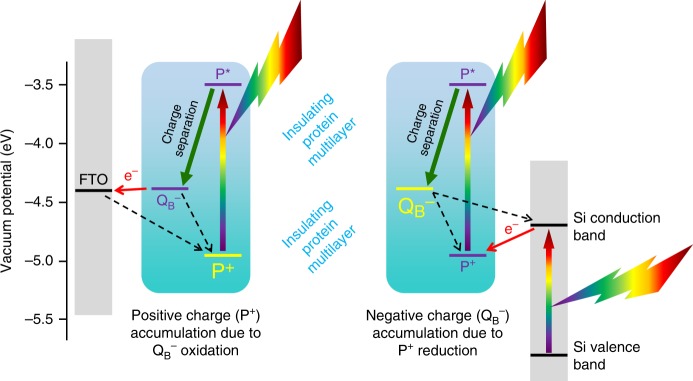
Photoexcitation and electrochemical activity at the two electrodes. In cells with RC-LH1 proteins (i.e., reaction centre-light harvesting 1 complexes), in the photo-excited protein layers near the FTO (fluorine-doped tin oxide) electrode (left), trapped positive charges (initially P^+^) accumulate as Q_B_^−^ formed by intra-RC charge separation (green arrow) donates electrons to the FTO (red arrow). In photo-excited protein layers near the n-Si electrode (right), trapped negative charges (initially Q_B_^−^) accumulate as P^+^ formed by intra-RC charge separation (green arrow) is reduced by the photoactive n-Si electrode (red arrow). In the absence of an electrolyte, these protein/electrode interactions cause the build-up of a potential difference over time. At either electrode, the process responsible for the generation of trapped positive or negative charges would be expected to be in competition with wasteful competing reactions (dashed black arrows)

Taking into account vacuum potentials, and a well-established understanding of the photochemistry of RC-LH1 complexes^25,26^, the observed photovoltage is attributed to net oxidation of RC-LH1 proteins at the FTO electrode (Fig. 3, left) and net reduction of RC-LH1 proteins at the n-Si electrode (Fig. 3, right), producing trapped charges on opposite sides of the protein multilayer. The initiating event, in either case, is the photogeneration of the radical pair P^+^Q_B_^−^ (Fig. 1c), which has a lifetime of 3–5 s in purified RC-LH1 complexes^38–40^, and which relaxes by recombination at the P BChls. Net oxidation of protein at the FTO-glass electrode would be achieved if Q_B_^−^ in a suitably oriented RC-LH1 protein is able to reduce the FTO more rapidly than either P^+^Q_B_^−^ recombination or reduction of P^+^ by the FTO (Fig. 3, left). At the photoactive n-Si electrode (Fig. 3, right), a net reduction of the adjacent protein would be possible if electrons from the conduction band of the n-Si were able to reduce P^+^ more rapidly than either P^+^Q_B_^−^ recombination or donation of an electron to the conduction band of the n-Si by Q_B_^−^.

The observed gradual build-up of the photovoltage (Fig. 2c) indicated that the density of trapped charges increased over several hundred seconds. This can be attributed to multiple oxidation or reduction events within individual complexes and/or propagation of trapped electrons or holes deeper into the multilayer through slow interprotein electron transfer. Regarding the former, it has been estimated from electrochemical titrations that an LH1 ring can store up to eight BChl cations without undergoing irreversible photo-oxidative damage^41^, and multiple negative charges can be accumulated on the quinone and bacteriochlorin cofactors of the RC when the rate of reduction of the photo-oxidized RC by an external donor exceeds the rate of charge recombination or quinone oxidation by an external acceptor^42,43^. In addition, RCs contain a single dissociable Q_B_ ubiquinone that can undergo double reduction, the second reduction being accompanied by double protonation, and preparations of RC-LH1 complexes typically contain 10–15 molecules of ubiquinone in the space between the RC and the surrounding LH1 ring that can exchange with the quinone at the Q_B_ site^38^. As a result it is plausible that a single RC-LH1 complex could accumulate more than one positive or negative charge if adjacent to an electrode that can act as an acceptor/donor. Regarding charge propagation, as RCs undergo charge separation under illumination, each can be treated as an electric dipole. In an ideal scenario where all dipoles were aligned with positive ends facing one electrode and negative ends facing the other, charge storage in the device could be possible without any transport mechanism in the active layer. However, since such a device configuration with perfectly aligned dipoles is unrealistic for proteins assembled into a multilayer, charge storage in the device is likely to also rely on the transport of charges between complexes in those layers closest to the surface of either electrode. It is highly probable that interprotein interfaces in the device act as charge localization sites allowing charges to hop from one state to other before they reach the electrodes. Incoherent hopping of charge carriers from site to site is known to be a dominant transport mechanism in disordered systems and has also been studied previously in photosynthetic systems^44–46^. Such gradual penetration of long-lived trapped charges into the photoactive protein multilayer through electron hopping between adjacent proteins could also be a factor in the slow discharge of the photovoltage on terminating the illumination period. (Fig. 2d). Although not of the same nature as the capacitive origin of voltage build-up in dry protein multilayers in this device, trends of slow photoresponse have been previously observed in liquid biophotoelectrochemical cells despite the addition of electron transport mediators^29,47–49^, where slow rise and decay in photovoltage were the direct effect of slow electrolyte diffusion^47^.

It should be noted that while charge transport could play an important role in the build-up of a charge difference between electrodes, it is crucial that long-range transport from one electrode to the other is avoided as this would counteract the charge storage ability of the BPC. In an ideal dielectric capacitor, there will not be any charge transport through the device and the charge storage is achieved purely by the accumulation of charges at the electrode/dielectric interface. In the BPCs the protein layers in the center of the device therefore serve the function of an insulating layer that enables the build-up of charge at the electrode/protein interfaces.

A factor in the use of RC-LH1 complexes as a material for charge storage is that it is known from experiments employing conductive atomic force microscopy that individual complexes conduct electrical current under an applied bias^50–52^. Such experiments typically interrogate a monolayer of protein oriented on a conductive surface, and it has been suggested that electron tunneling across an LH complex is facilitated by the carotenoid cofactors^50–52^. However, multilayers of these proteins, arranged randomly with respect to an applied bias, would not be expected to conduct electrical current over a long range. Given this, a possible reason that the photovoltage supported by an RC-LH1 multilayer increased as its thickness increased up to 500 µm (Fig. 2b) is that the effective resistance of the multilayer also increased, enabling the build-up of a larger potential difference. Such an explanation seems more plausible than positing that thicker layers produced higher voltages because they were more absorbing, as photochemical activity leading to trapped charges would be expected to be confined to a minority of the structure comprising (probably several) protein layers close to either electrode, rather than the entire thickness of the multilayer. The decrease in photovoltage achieved by films thicker than 500 µm (Fig. 2b) is attributed to poor light penetration to the n-Si back electrode and its adjacent RC-LH1 proteins that lessens the accumulation of negative charge at this interface.

**Fig. 4:**
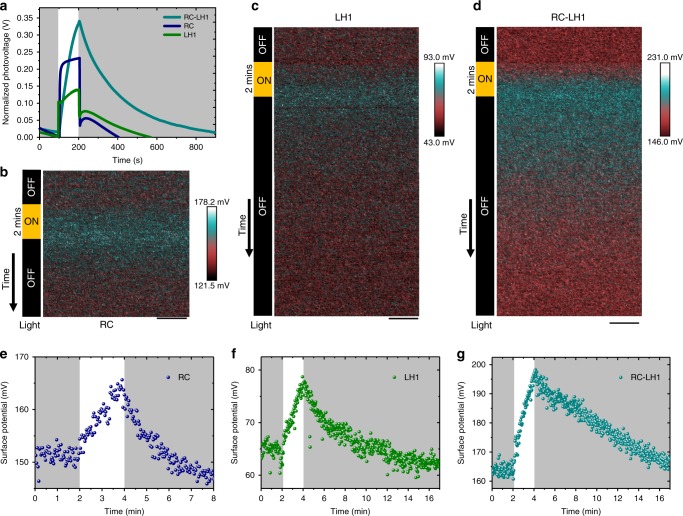
Photocharging and dark discharging of photoprotein films. **a** Bulk charge–discharge characteristics of 500 μm films of three different photoproteins namely, reaction centres (RC), light harvesting 1 complexes (LH1) and reaction centre-light harvesting 1 complexes (RC-LH1), exposed to 100 s illumination. **b**–**d** KPFM (Kelvin-probe force microscopy) surface potential maps of **b** RC, **c** LH1, and **d** RC-LH1 films before, during and after a 2 min period of illumination; scale bars denote 200 nm. **e**–**g** Changes in surface potential before, during and after a 2 min period of illumination of **e** RC, **f** LH1, and **g** RC-LH1 films

The *Rba. sphaeroides* RC-LH1 photosynthetic complex is modular (Fig. 1b–d), and the RC and LH1 proteins can be purified as separate, functional entities (absorbance spectra shown in Supplementary Fig. 1). BPCs formed from isolated RCs or LH1 complexes also generated a photovoltage (Fig. 4a). On comparing charge–discharge characteristics for 500 μm protein films, it was found the photovoltage reached at the end of a standard 200 s illumination was the highest for the combined RC-LH1 complex and lowest for the LH1 protein (Fig. 4a). The light harvesting capacity of isolated RCs is limited to its six bacteriochlorins and one carotenoid (Fig. 1c), but each RC-LH1 complex has an additional 32 BChl and 32 carotenoid light-harvesting pigments (Fig. 1b, d). The lower photovoltage achieved by a film of RCs is therefore likely attributable to a strongly diminished light harvesting capacity even though, as they are smaller than an RC-LH1 complex, the concentration of RCs in a packed multilayer would be expected to be greater. Although the pigment content of the isolated LH1 antenna protein is closer to that of RC-LH1 complexes, the absence of the RC means that light harvesting is not translated into a meta-stable charge separation, but rather energy is lost as emission and heat. Nevertheless, a photovoltage is still obtained because LH1 is capable of acting in a manner akin to an organic semiconductor, injecting excited electrons into the FTO electrode and becoming positively charged, or receiving electrons from the n-Si electrode and becoming negatively charged (Supplementary Fig. 2). However, the number of photo-accumulated charges is likely lower than in RC or RC-LH1 complexes because the lifetime of the LH1 excited state (estimated as 680 ps at room temperature^53^) is very short relative to the 1–5 s lifetime of the charge-separated state formed in RC and RC-LH1 complexes.

### Kelvin-probe microscopy to assess potential build-up and decay

Charging and discharging of the different types of BPC were further investigated by recording the surface potentials of the three types of protein film by KPFM. Films coated on an FTO substrate were scanned using a conductive Pt/Ir probe before, during and after illumination. For all three films, the surface potential measured along a 1000 nm trajectory increased after light-on and decayed gradually after light-off (Fig. 4b–d), confirming the generation of a photovoltage. In good accord with the trend in overall device photovoltage (Fig. 4a), the averaged surface potential shift (Fig. 4e–g) was highest for the RC-LH1 films (~30 mV) and lower for LH1 or RC films (~15 mV). The time for relaxation of the surface potential shift was also longest for RC-LH1 films (~15 min) and shortest for RC films (~3 min) (Fig. 4e–g). These relaxation times of minutes confirmed that the potential shifts were due to the photogeneration of trapped charges rather than photochemistry within individual proteins, where relaxation occurs in a few seconds.

**Fig. 5:**
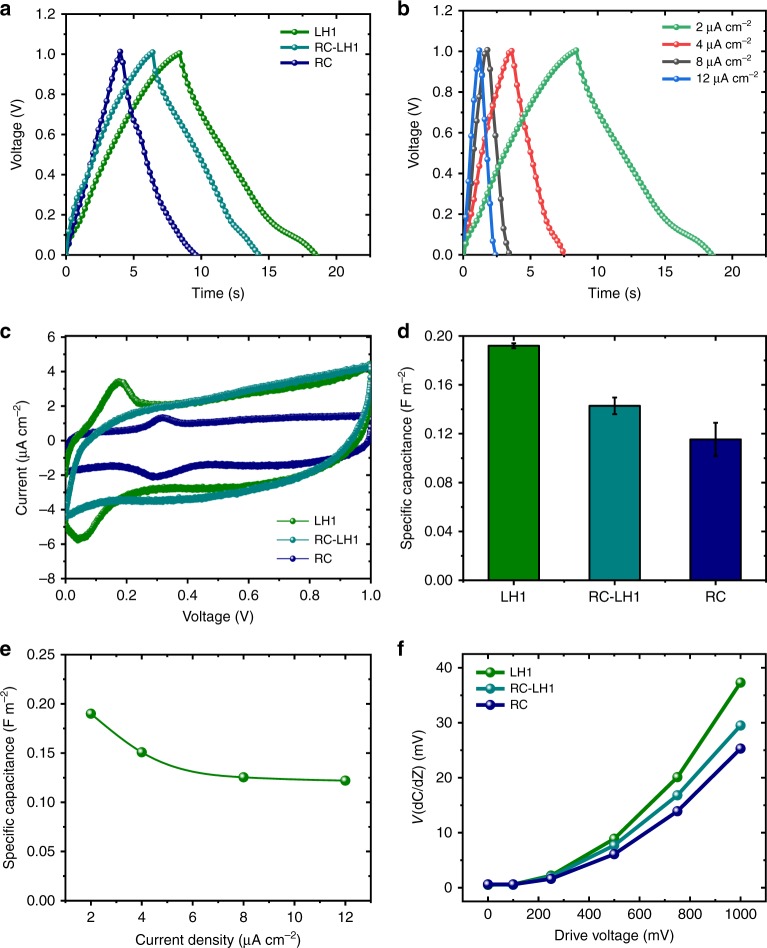
Capacitances of the biophotonic power cells. **a** Galvanostatic charge–discharge over a 0–1 V range for biophotonic power cells (BPCs) with three different photoproteins namely, reaction centres (RC), light harvesting 1 complexes (LH1), and reaction centre-light harvesting 1 complexes (RC-LH1), at an applied current density of 2 μA cm^−2^. **b** Galvanostatic charge–discharge curves for an LH1 BPC at different current densities. **c** Cyclic voltammetry curves for the three BPCs. **d** Specific capacitance of the three BPCs; capacitance presented is the average of at least three measurements and the error bar denotes standard deviation. **e** Specific capacitance of an LH1 BPC at different applied current densities. **f** Capacitance gradient *V*_(dC/dZ)_ as a function of drive voltage for the three types of cell

The KPFM and macroscopic photovoltage measurements indicated that all three proteins can generate and store charge. However, the extent of charge accumulation by each protein under these conditions was determined by its light-harvesting capacity and photochemical activity rather than its innate charge storage capacity. As a result, the discharge times in Figs. 2 and 4 did not necessarily convey the relative charge storage capacities of the three proteins as each was charged to different extent under illumination. To investigate the capacitance of each of the proteins at a macroscopic level, galvanostatic charge–discharge measurements and CV were performed on the two electrode devices (Fig. 5a–f). Near-symmetrical charge–discharge responses were obtained over a 0–1 V voltage window at an applied current of 2 µA cm^−2^, indicating a good capacitive behavior (Fig. 5a). The LH1 photoprotein exhibited the largest area under the curve, indicating the highest capacitance, followed by RC-LH1 and then RC. Galvanostatic charge–discharge responses of the LH1 cell over a range of applied currents from 2 to 12 µA cm^−2^ were also near-symmetrical, confirming the capacitive behavior (Fig. 5b).

### Microscale and macroscale capacitance measurements

CV confirmed the higher capacitance of the LH1 cells compared to the RC-LH1 and RC cells, the area of the near-rectangular CV plot being the highest for LH1 (Fig. 5c, and see Supplementary Fig. 3). It is known that the capacitance is proportional to the area under the CV curve (∫*iv*—where *i* and *v* are the current and applied potential, respectively). Using the galvanostatic charge–discharge responses, the specific capacitance can be determined from the applied current, the active area and the slope of the discharge curve. From the charge–discharge profiles the specific capacitance was found to be the highest for LH1 films (≈0.19 F m^−2^), followed by RC-LH1 films (≈0.14 F m^−2^) and RC films (≈0.11 F m^−2^) (Fig. 5d). Over a 2–12 µA cm^−2^ range of applied current, the LH1 cells exhibited a maximal capacitance at 2 µA cm^−2^ and the capacitance did not drop beyond 0.12 F m^−2^ over the entire range (Fig. 5e).

These differences in capacitance were confirmed on the microscopic level using SCM measurements in which the same amount of injected charge was made available to each type of protein film by applying a range of drive voltages from an external source. The determined gradient of capacitance between the AFM tip and the sample, *V*_(dC/dZ)_, is an indirect measure of the dielectric constant of the sample. SCM maps were recorded over a defined area of RC-LH1, LH1, or RC film at six different drive voltages and converted into average values of *V*_(dC/dZ)_ to establish uniformity (Supplementary Fig. 4). A plot of the capacitance gradient averaged across the scanned film area as a function of drive voltage (Fig. 5f) showed that *V*_(dC/dZ)_ was highest for LH1 films and lowest for RC films at each drive voltage, in agreement with the trend in specific capacitances derived from charge–discharge and CV measurements (see above). Despite these measured capacitances exhibiting the order LH1 > RC-LH1 > RC, the observed photovoltage displayed the trend RC-LH1 > RC > LH1, consistent with the RC-LH1 complex being superior to RCs in light harvesting and superior to LH1 complexes in the translation of short-lived excited states into long-lived charge-separated states.

### Proof-of-concept demonstration and outlook

**Fig. 6:**
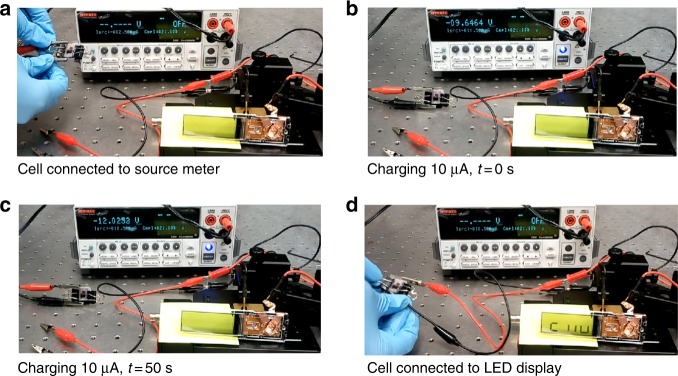
Storage of externally injected charges by a single biophotonic power cell. **a** A single-biophotonic power cell (BPC) with RC-LH1 (reaction centre-light harvesting 1 complexes) was connected to source meter. **b** Charge was injected into the cell by applying a constant current of 10 μA. **c** The cell was charged for 50 s. **d** The charged cell powered an LED display for approximately 1 s

Finally, the utility of the 500 µm RC-LH1 BPCs was demonstrated through their ability to power a low-consumption light emitting diode (LED) display. In the demonstration depicted in Fig. 6, a single RC-LH1 cell was charged by a constant current of 10 μA for 50 s (Fig. 6a–c). This provided sufficient charge to power an LED display for approximately 1 s (Fig. 6d). In a second experiment of this type (Supplementary Fig. 5) three RC-LH1 cells connected in series were charged by a 1 mA current for 2 s and were able to power an LED display for up to 5 s. Photo-charging of a bank of four RC-LH1 cells at 1 sun illumination for 5 min powered an LED display for about a second (Supplementary Fig. 6). The mode of operation of the BPCs in these charging demonstrations can be best understood in terms of a parallel-plate capacitor in which the BPC plays a dual role of capacitor and, when illuminated, an integrated power source (Supplementary Fig. 7)

## Discussion

In summary, we show with microscopic and macroscopic evidences that the purple bacterial photoprotein multilayers exhibit prolonged charge storage. The phenomenon unveiled not only casts new light on the photonic and electronic properties of the photosynthetic proteins but also presents a promising application in energy research. Looking to future developments, a key feature of the BPCs described in this report was the use of protein multilayers to generate the photovoltage and store charges. As these films were fabricated by simply drop-casting a concentrated solution of detergent-solubilized protein into a pre-formed well it is to be expected that individual proteins had a random orientation within the multilayer. One way to possibly boost the photovoltages obtained may be to control the orientation of individual RC-LH1 proteins throughout the multilayer such that there is alignment of the dipoles created between the P and Q_B_ termini of the RC on photoexcitation. Large photovoltages obtained from micron thick crystals of the Photosystem I (PSI) RC from *Pisum sivatum* (pea) in response to strong (1.1 W cm^−2^) 660 nm laser excitation have been attributed to a superposition of photoinduced dipoles across uniformly oriented PSI proteins within the crystal lattice^54,55^. In the present case, imposing a uniform alignment on the RC-LH1 complexes within a film could both maximize desired redox interactions at each electrode surface (i.e., Q_B_ oxidation at the FTO-glass anode or P^+^ reduction at the n-Si cathode) and align the dipoles created across individual RC-LH1 complexes, both of which could potentially lead to a higher photovoltage. However, it is worth noting that achieving such control over protein orientation in a thick, multilayer film is challenging, and an attractive feature of the unoriented protein films used in the present work is their simplicity of fabrication.

## Methods

### Biological materials

Integrated power cells were prepared using either the PufX-deficient RC-LH1 complex from the purple photosynthetic bacterium *Rba. sphaeroides* or the component RC or LH1 proteins. These were isolated from three strains of *Rba. sphaeroides* in which either RC-LH1, RC, or LH1 was the sole pigment–protein in the cell^30,31,56^. Bacterial cells were grown in M22 + medium under dark/semiaerobic conditions^57^. His-tagged RCs were solubilized from photosynthetic membranes using 1.5% (w/v) n-dodecyl-N,N-dimethylamine-N-oxide (LDAO) and purified by nickel affinity chromatography on a FF Ni-NTA column (GE Healthcare), followed by size exclusion chromatography on a Superdex 200 16/60 column (GE Heathcare)^56^. RCs were exchanged into 20 mM Tris (pH 8)/0.04% n-dodecyl β-D-maltopyranoside (DDM) during size exclusion chromatography and concentrated in this buffer to an absorbance at 802 nm (A^802^) of ≈350 absorbance units. RC-LH1 complexes which had a His-tag on the RC component were isolated from photosynthetic membranes using 2% (w/v) (DDM) and also purified using nickel affinity chromatography on a FF Ni-NTA column (GE Healthcare) followed by size exclusion chromatography on a Superdex 200 16/60 column (GE Heathcare)^58^. RC-LH1 complexes were concentrated in 20 mM Tris (pH 8)/0.04% DDM to an A^875^ of ≈600 absorbance units. For LH1 complexes, cells from 3 L of culture medium were harvested by centrifugation and resuspended in 100 mL of 20 mM Tris (pH 8), containing several crystals of DNAseI. Cells were lysed at 20,000 psi in a Constant Systems cell disruptor and cell debris was removed by centrifugation at 27,000 × *g* for 15 min at 4 °C. The supernatant was loaded on 15/40% (w/v) sucrose step gradients made up in 20 mM Tris (pH 8.0), and the gradients were ultracentrifuged at 113,000 × *g* and 4 °C for 2 h. Membranes were harvested from the interface, diluted with 20 mM Tris (pH 8.0) and pelleted by ultracentrifugation at 113,000 × *g* and 4 °C for 2 h. The membrane pellet was then resuspended overnight in 20 mM Tris (pH 8.0) to an optical density at 875 nm of approximately 40 cm^−1^. LH1 was isolated from membranes by the addition of 3.0% octyl glucoside (OG) followed by stirred incubation in the dark at room temperature for 90 mins. Membrane debris was removed by centrifugation at 113,000 × *g* for 30 min at 4 °C. The supernatant was passed through 3 × 5 mL DEAE anion exchange columns (GE Heathcare) that had been equilibrated with 75 mL of 20 mM Tris (pH 8.0)/0.1% OG. The columns were washed with 150 mL of 20 mM Tris (pH 8.0)/0.1% OG/150 mM NaCl and bound protein eluted with 50 mL of 20 mM Tris (pH 8.0)/0.1% OG/300 mM NaCl. Eluted protein was further purified using a Superdex 200 16/60 column (GE Heathcare) pre-equilibrated in, and run with, 20 mM Tris (pH 8)/0.04% DDM to achieve detergent exchange from OG to DDM. Fractions with a ratio of absorbance at 875 nm and 280 nm (A^875^/A^280^) above 2.0 were pooled and concentrated using a 100 kDa cut-off spin concentrator (Amicon) until the A^875^ was at least 600 cm^−1^. All purified proteins were aliquoted and stored at −80 °C until use.

### Experimental design and device construction

Aliquots (20–1000 µL) of concentrated protein solution were drop-cast and vacuum dried onto FTO-glass in a Parafilm well that had an area of 0.05 ± 0.005 cm^2^ and a uniform depth varying between 0.1 and 2 mm. Once the protein layer was dried a precleaned n-Si counter electrode was placed on the well and held in place using binder clips.

### Device-photovoltage measurements

All photovoltage measurements were carried out using a Keithley K2400 source meter under white light excitation at an intensity of 100 mW cm^−2^ approximating to 1 sun illumination.

### Microscopic studies on charge build-up and decay

KPFM was carried out using a Dimension ICON microscope (Bruker Nano Surface, Santa Barbara, CA). Amplitude modulation was used to obtain a high signal to noise ratio, and all measurements were performed in a dual pass mode to eliminate any topography effects. Scans were carried out using Pt/Ir coated SCM-PIT probes with an applied bias and the surface potential of the protein film determined from the contact potential difference (CPD) between the tip and the film. As the probe traversed the sample scan area the film was illuminated for 2 min using a tungsten-halogen lamp at an incident light intensity of 10 mW cm^−2^ and surface scanning was continued during the subsequent dark period until the base surface potential was reached. For scans in which the base potential did not reach the original level after illumination was turned off within the scan range of 0–1000 nm, the scan was instantly continued in the same scan area (from the 1000 to 0 nm position) until the original base potential was reached. This produced scan maps for the RC-LH1 and LH1 films that were twice the area of those for the RC. For SCM the gradient of capacitance between the probe and the sample, *V*_(dC/dZ)_, was measured to give an indirect measure of the capacitance of the protein film. All KPFM and SCM measurements were performed on 50 µm thick protein films drop cast onto FTO glass. Thicker protein films were not used because they had a high surface roughness that resulted in high noise in the potential/capacitance scans. The illumination intensity 10 mW cm^−2^ was the maximum achievable using a bespoke illumination setup.

### Device-capacitance measurements

CV and galvanostatic charge/discharge measurements were conducted using Solartron System 1470E. Capacitance was quantified from the charge–discharge patterns and the estimated capacitance could be attributed mainly to the proteins, neglecting the capacitance resulting from the surrounding paraffin film. The paraffin film, which had a dielectric constant in the range of 1.9–2.5, resulted in a negligibly low capacitance in the range of a few pF which was 5 orders of magnitude lower than the overall device capacitance.

## Supplementary information

Supplementary Information

## Data Availability

All relevant data will be made available upon reasonable request. Requests for data should be addressed to S.C.T.
