# Supplementary material for: Photosynthetic apparatus of Rhodobacter sphaeroides exhibits prolonged charge storage
Source: Nat Commun. 2019 Feb 22;10:902. doi: 10.1038/s41467-019-08817-7 (PMC6385238; doi:10.1038/s41467-019-08817-7)
Supplement: Supplementary file 1 — Supplementary Information [file 41467_2019_8817_MOESM1_ESM.pdf]

Photosynthetic apparatus of *Rhodobacter sphaeroides* exhibits prolonged charge storage

Supplementary Information

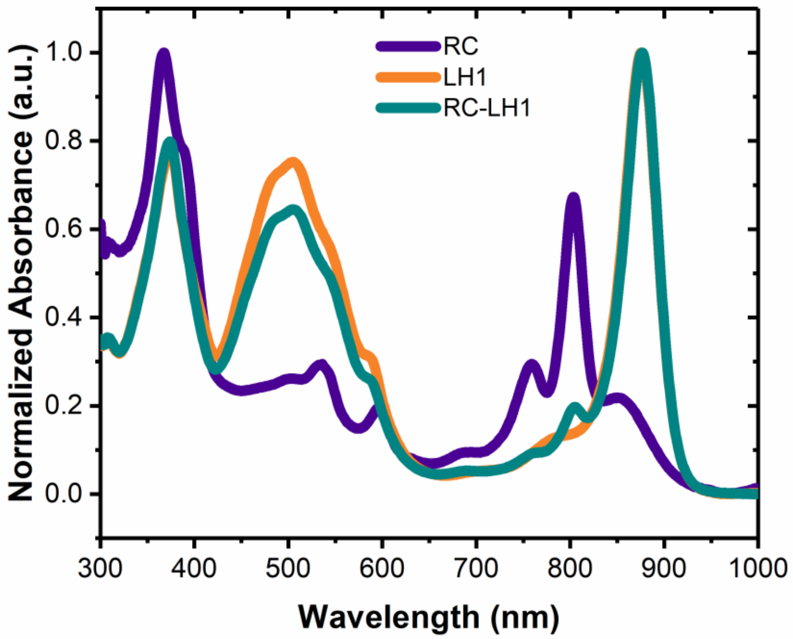

Supplementary Figure. 1: Absorption characteristics of the protein complexes. Normalized absorption spectra of RC, LH1 and RC-LH1 complexes in solution. The LH1 complex shows a major absorbance bands at 875 nm and 375 nm and a minor band at 600 nm arising from the LH1 bacteriochlorophylls and bands between 400 and 550 nm arising mainly from the LH1 carotenoids. In addition to an equivalent 360 nm bacteriochlorophyll band the RC displays three bacteriochlorin absorbance bands between 700 and 950 nm. The absorbance spectrum of the RC-LH1 complex is a composite of these two spectra, for example the major band at 875 nm being attributable to the LH1 domain and the minor band at 805 nm being attributable to the RC.

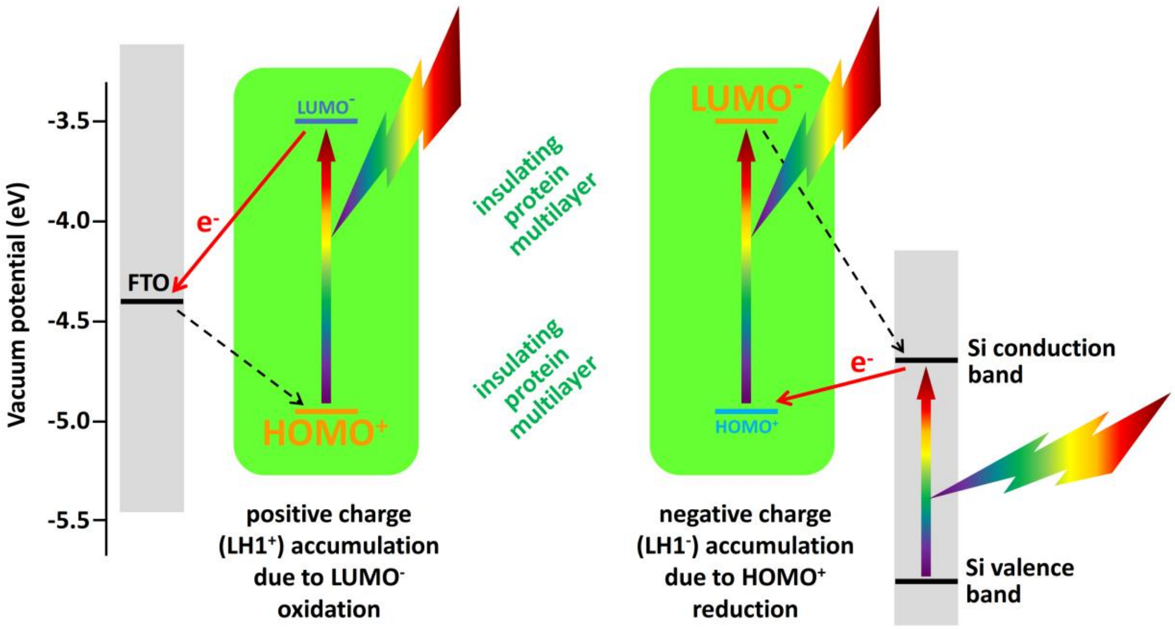

Supplementary Figure. 2: Photoexcitation and electrochemical activity at the two electrodes in an LH1 BPC. In photo-excited LH1 layers near the FTO electrode (left), trapped positive charges accumulate as excited state electrons are donated to the FTO (red arrow). In photo-excited LH1 layers near the n-Si electrode (right), trapped negative charges accumulate as photo-excited LH1 is reduced by the photoactive n-Si electrode (red arrow). In the absence of an electrolyte, this causes build-up of a potential difference between electrodes. At either electrode, the process responsible for generation of trapped positive or negative charges would be expected to be in competition with competing reactions (dashed black arrows).

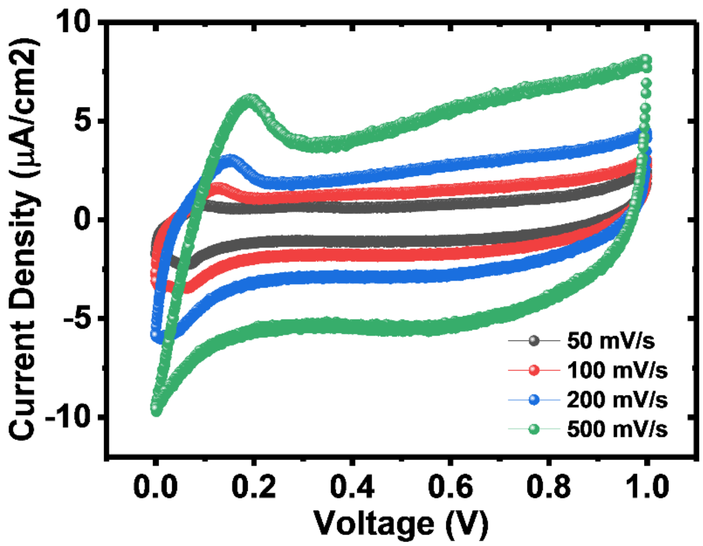

Supplementary Figure. 3: Cyclic voltammetry (CV) of LH1 BPC. CV curves at scan rates of 50, 100, 200 and 500 mV/s for an LH1 BPC.

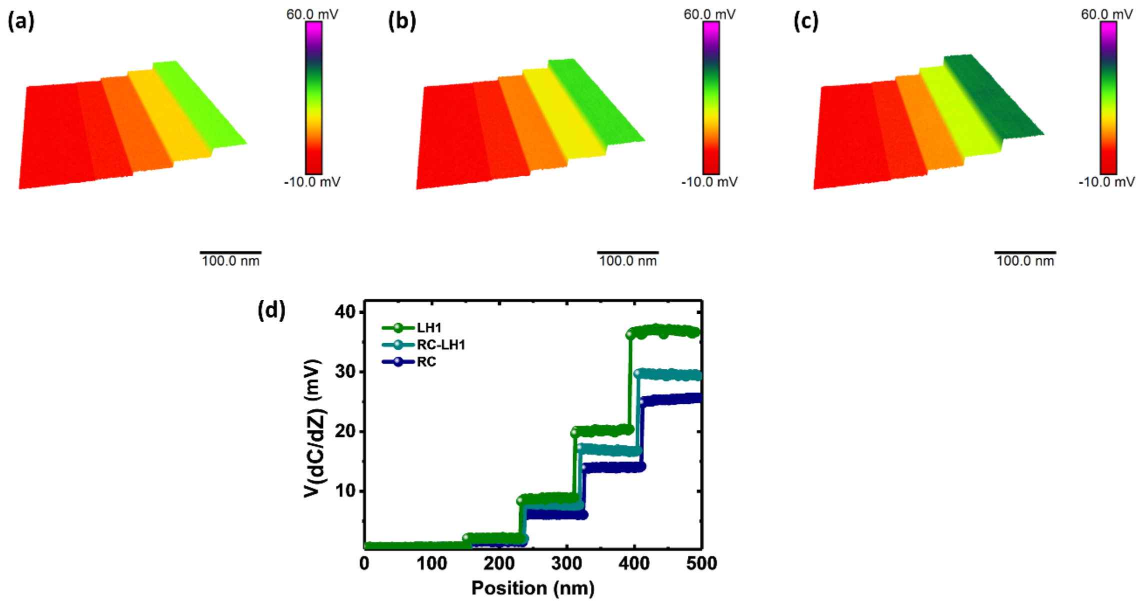

Supplementary Figure. 4: Scanning capacitance microscopy (SCM) maps. Data are shown for films formed from (a) RC, (b) RC-LH1 and (c) LH1 complexes. Each step in the map corresponds to an applied drive potential of (left to right) 0 mV, 100 mV, 250 mV, 500 mV, 750 mV and 1000 mV. (d) Plot of capacitance gradient as a function of horizontal position for the three films.

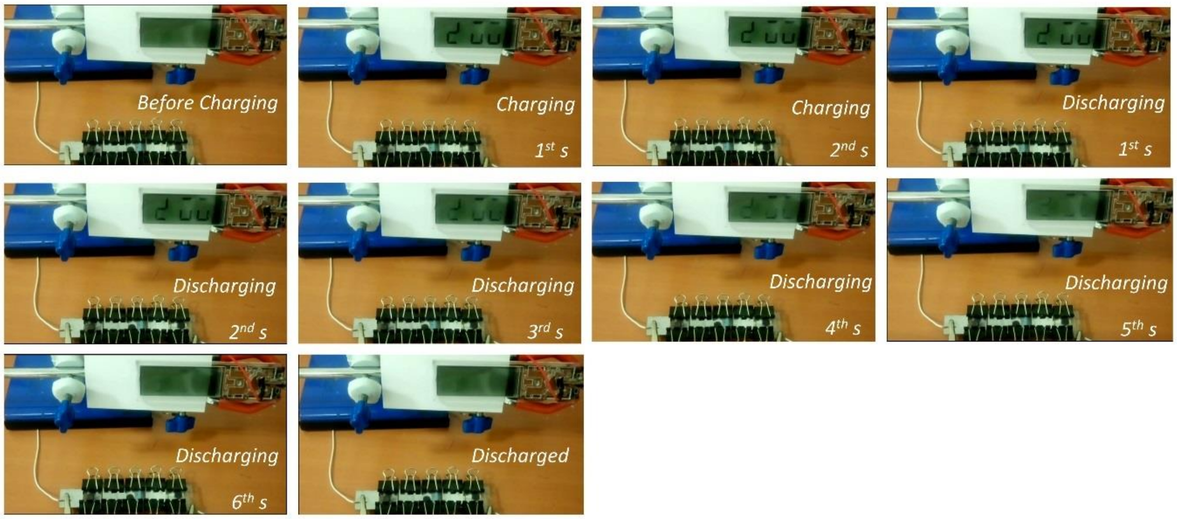

Supplementary Figure. 5: Storage of externally injected charges by a bank of three RC-LH1 cells. Three RC-LH1 cells were connected in series and then were connected to a source meter and the two end-terminals of the cells were connected to the LED display (top left). Charges were injected into the cell by applying a constant current of 1 mA for 2 seconds during which the LED display was powered, but still under the control of the power source (top centre). When the current input was then turned off (top right), the cells were allowed to discharge by powering the LED display connected in the circuit. The LED display was powered for up to 5 s while gradually fading off (remaining panels).

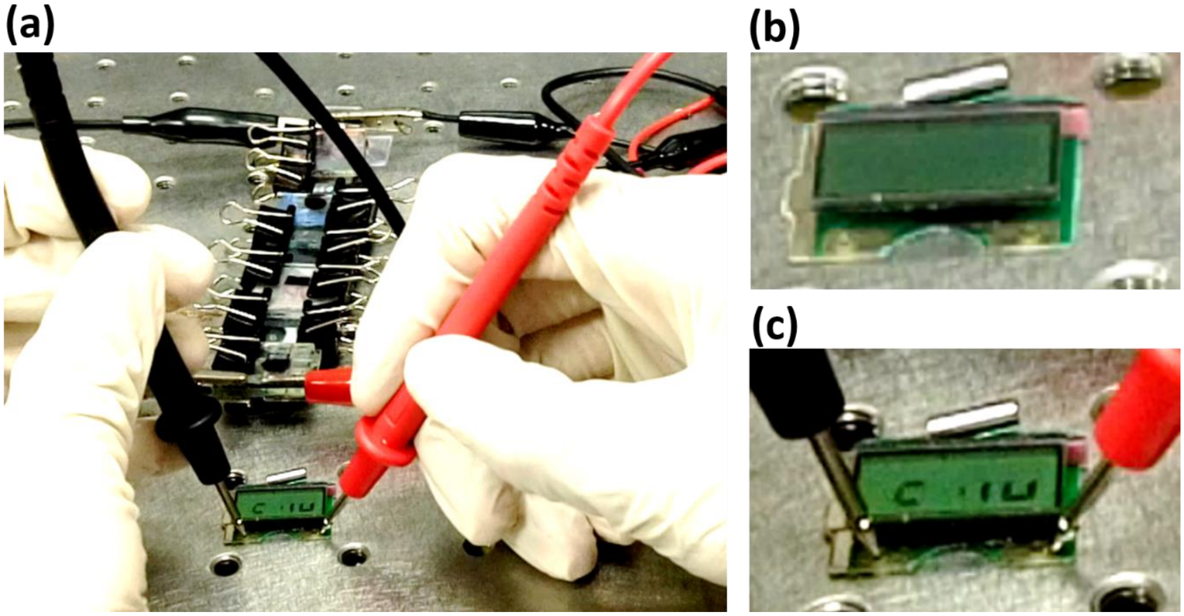

Supplementary Figure. 6: Storage of photo-generated charges by a bank of four RC-LH1 cells. (a) Four RC-LH1 cells connected in series were exposed to 1 sun illumination for 5 mins. The cells were then connected to an LED display. The cell electrodes had static accumulated charges that surged into the display terminal as a lead from the cells comes in contact, which resulted in a short-lived current that powered the LED display for about a second, discharging the cells; (b) LED display before connecting the cells. (c) LED display after connecting the cells.

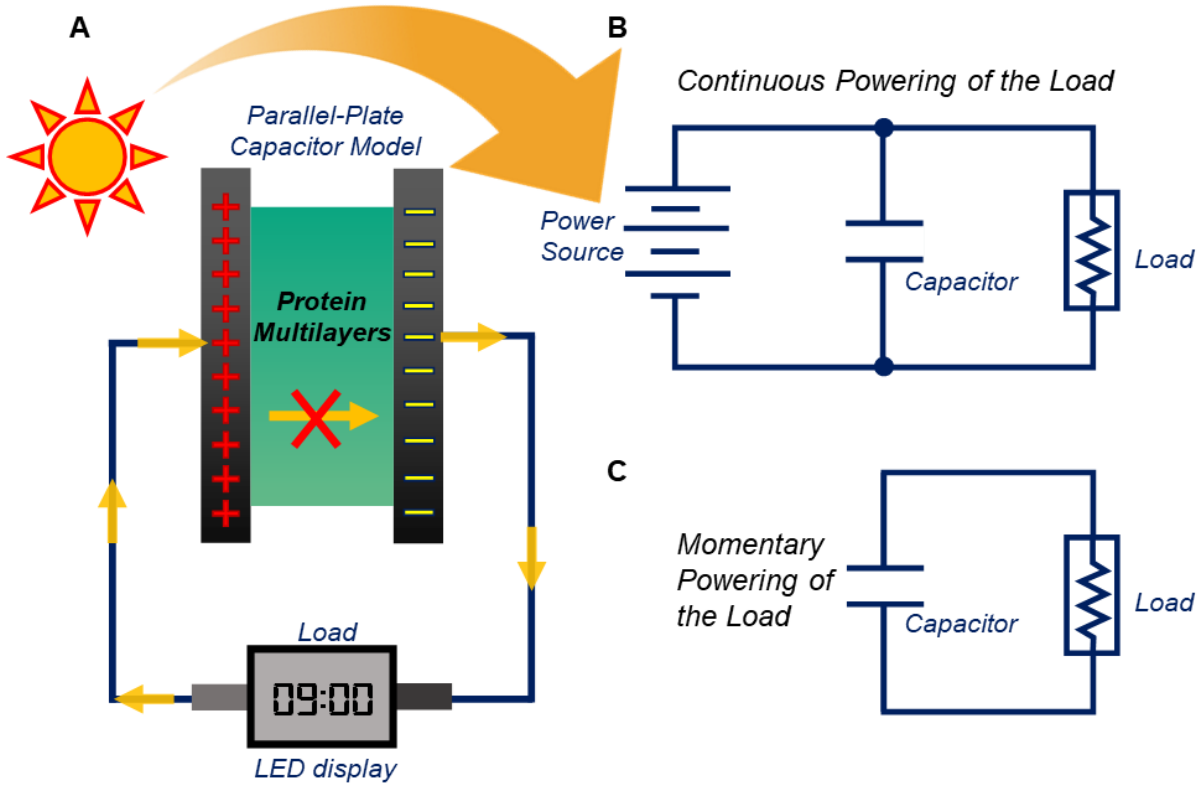

Supplementary Figure. 7: Mode of operation of a BPC in an electrical circuit: (A) The BPC is analgous to a parallel-plate capacitor. As with any typical capacitor, no direct current (DC) can/should pass through the device. However, continuous powering of a load is still possible as the circuit is completed by the flow of electrons in the external circuit; the electrons from one plate of the capacitor reach the opposite plate through the load which gradually brings the potential difference between the two plates to zero. (B) When a BPC under illumination is connected to a load, the circuit formed is analogous to a resistor/capacitor (RC) circuit connected in parallel to a power source, with light acting as the power source for the BPC. In such a circuit, the load can ideally be indefinitely powered. (C) When a pre-charged BPC in the dark is connected to a load, the circuit would be analogous to an RC circuit without any power source; In this case, while the delivery of power is still continuous, it is not indefinite and is limited by the amount of charge stored in the capacitor.
